# Longevity in plants impacts phylogenetic and population dynamics

**DOI:** 10.1111/nph.70654

**Published:** 2025-10-15

**Authors:** Stephen A. Smith, James B. Pease, Tom Carruthers, Gideon S. Bradburd, Indah B. Huegele, Gregory W. Stull, William N. Weaver, Yingying Yang, Ting‐Shuang Yi, Jeremy M. Beaulieu

**Affiliations:** ^1^ Department of Ecology and Evolutionary Biology University of Michigan Ann Arbor MI 48109 USA; ^2^ Department of Evolution, Ecology and Organismal Biology The Ohio State University Columbus OH 43210 USA; ^3^ Idaho Museum of Natural History Idaho State University Pocatello ID 83209 USA; ^4^ Department of Botany, National Museum of Natural History Smithsonian Institution Washington DC 20560 USA; ^5^ Kunming Institute of Botany Chinese Academy of Sciences Kunming Yunnan 650201 China; ^6^ Department of Biological Sciences University of Arkansas Fayetteville AR 72701 USA

**Keywords:** agent‐based models, life history, longevity, phylogeny, simulations, substitution rates

## Abstract

- Phylogenies of long‐lived plants often exhibit short molecular branch lengths and high levels of gene‐tree conflict. However, the biological mechanisms underlying these patterns remain unclear. We examine this with simulations and through empirical examination of several large seed plant clades.
- We used an agent‐based simulation model varying lifespan, degree of overlapping generations, and somatic mutation. We also compared simulated outcomes to phylogenomic patterns in several datasets of seed plants that include life‐history shifts.
- Lifespan and overlapping generations together can generate both short branches and elevated gene‐tree conflict. Somatic mutation can amplify these effects, although available evidence suggests mutation rates are often too low to drive major phylogenetic consequences. Variation across simulation parameterizations can mirror the diversity of phylogenomic patterns observed among lineages with differing life histories.
- Lifespan and generation overlap are potentially major contributors to characteristic phylogenetic signatures in long‐lived plants. Consequently, life history should be considered when interpreting evolutionary patterns, substitution rates, and among‐lineage heterogeneity in long‐lived plant lineages.

## Abstract 2

See also the Commentary on this article by Althaus & Hipp, **250**: 12–14.

## Introduction

*I can't compare with it; and I've known some ships made of dead trees outlast the lives of man made of the most vital stuff of vital fathers*. Chapter 135 The Chase ‐ Third day, Moby Dick, Herman Melville

Life histories across plants range from the ephemeral to the effectively eternal. In natural populations, beech trees (*Fagus*) set their first seeds after 40 yr and can live for hundreds of years; *Arabidopsis* individuals live their entire life within 6–12 wk. Variation in life histories can cause markedly different selection pressures leading to dramatically different adaptations (Salguero‐Gómez *et al*., 2016; Brooks *et al*., 2024). For example, a 1000‐yr‐old oak will likely experience the upheaval of several large‐scale climate events while concurrently subject to the tides of seasonal variation.

Diverse life‐history strategies and evolutionary transitions can pose substantial challenges to phylogenetic inference (Smith & Donoghue, 2008; Lanfear *et al*., 2013). Smith & Donoghue (2008) demonstrated that substitution rates were slower for woody lineages and discussed longevity as a potential cause. Expanding on earlier studies primarily focused on a few ‘neutrally evolving’ gene or spacer regions, Yang *et al*. (2023) demonstrated that substitution rates were lower for longer‐lived species than shorter‐lived species across thousands of genes within the Caryophyllales. Lanfear *et al*. (2013) showed that life‐history traits, including generation time, woodiness, and height, are significantly associated with substitution rates across angiosperms. Smith and Beaulieu (2009) further demonstrated associations with molecular rate and climate evolution. Beaulieu *et al*. (2010) found that rates of genome size evolution also vary with growth form, suggesting that life history can influence molecular evolution at broader genomic scales. Beaulieu *et al*. (2015) also showed how life‐history variation in molecular rates can adversely impact divergence‐time estimates. Likewise, studies by Bromham (2009) and Bromham *et al*. (2015) explored how longevity and metabolic rate influence rate variation. Despite these and many other studies (e.g. Leitch & Leitch, 2012; Beaulieu *et al*., 2015), the specific aspects of life history that impact phylogenomic patterns remain unsettled.

One challenge of studying lifespan as an evolutionary parameter is that lifespan is indirectly connected to several other temporal life‐history parameters that may impact molecular rates. Maximum lifespan sets a ceiling on average lifespan, maximum generation time, average generation time, time to first reproduction, and average parental age. An increase in the variance of all these life‐history intervals is also expected as the maximum lifespan extends. This means that the potential for overlapping generations will tend to increase as lifespans increase and generations become shorter. We must also acknowledge the distinction between *maximum potential* lifespan and *average realized* lifespan. This is especially important for trees, for which intrinsic potential lifespans can be extremely long and can differ drastically from realized lifespan, which is largely determined by extrinsic mortality factors. Both lifespan and mortality influence survival and are governed by genetic systems that may be independent or have an underlying connection.

Somatic mutations present an additional challenge for studying the connection between life history and molecular rate, particularly in plants. Trees do not sequester their gametes, which creates the possibility that somatic mutations may accumulate throughout an individual's lifespan, meaning that longer‐lived individuals may carry a diversity of disparate genomes, potentially compensating for lower substitution rates of longer generation times (Burian *et al*., 2016; Schoen & Schultz, 2019; Hofmeister *et al*., 2020; Lesaffre, 2021). Research on somatic mutations in long‐lived organisms is limited, but a few notable exceptions have provided valuable insights. A 234‐yr‐old oak tree (*Quercus robur*) exhibited a surprisingly low number of somatic mutations (Schmid‐Siegert *et al*., 2017). Similarly, an investigation into the massive and ancient Pando aspen clone (*Populus tremuloides*) found modest spatial genetic structure, indicating localized mutation accumulation rather than widespread dispersal over its 16 000‐ to 18 000‐yr lifetime (Pineau *et al*., 2024). Tissue collected at multiple locations on the corpus of two individuals from different tropical tree species demonstrated that low‐frequency somatic mutations are heritable, challenging the assumption that only high‐frequency mutations contribute to evolution (Schmitt *et al*., 2024). Recently, Johannes (2025) suggests that the physical branching structure of trees may limit somatic mutations and their impact on mutation accumulation. While there are still relatively few studies that examine this issue extensively, they collectively highlight that heritable somatic mutations are relatively rare (Orr *et al*., 2020). Still, these mutations can be transmitted to progeny and contribute to population evolution.

Phylogenomic investigations of the past decade have deepened our understanding of long‐lived plant evolution. Large‐scale sequencing efforts have allowed for the analysis of hundreds, if not thousands, of gene trees and the complexities therein (One Thousand Plant Transcriptomes Initiative, 2019; Smith *et al*., 2020). One consistent pattern is that long‐lived plant species tend to have high rates of gene‐tree conflict. Conflict can be acute in long‐lived trees; it is generated both by slow substitution rates and short speciation intervals, which create patterns of incomplete allele sorting (i.e. hemiplasy) and by the prevalence of recent and ancient hybridization events (Folk *et al*., 2018; Pease *et al*., 2018; Zhang *et al*., 2021, Parins‐Fukuchi *et al*., 2021). For example, the Ericales includes lineages that live for more than a 1000 yr as well as annuals, such as *Primula* species, with their ancestors inferred to be long‐lived trees (Carruthers *et al*., 2024). Gene‐tree conflict in Ericales is so extensive that the ancestral relationships remain unresolved (Larson *et al*., 2020; Carruthers *et al*., 2024). Fagales includes long‐lived species, such as *Quercus* and *Fagus*, and also exhibits this pattern (Hipp *et al*., 2020). Given that the external causes of isolation and speciation occur similarly over time (e.g. climatic and geologic events), regardless of the organism's lifespan, differences in conflict patterns are expected between short‐ and long‐lived species due to the differential rates of substitution.

Here, we address how longevity and time to first reproduction affect population biology and phylogenetics. While traditional population genetics has produced many mathematically sound and powerful tools, there has been relatively less work on the impacts of overlapping generations (although see Felsenstein, 1971; Hill, 1972; Hill, 1972; Nunney, 1991; Nunney, 1993; Orive, 1993; Clark *et al*., 2024) and long life histories on substitution rates and phylogenetic conflict, and even less when somatic mutations are taken into account. We construct an agent‐based simulation to isolate potential causes of lowered substitution rates and increased conflict and then explore the relationship between conflict and effective population size. An agent‐based simulation allows us to construct a more realistic model of evolution in which individuals are discrete entities with heritable traits, subject to stochastic birth and death processes, thereby permitting explicit modeling of demographic fluctuations and genetic histories. In this context, we can examine how specific life‐history properties (maximum age, time to first reproduction, mutation rates, and death rate) impact substitution rates and phylogenetic conflict. In addition to this simulation, we examine several empirical cases in more detail to better understand the aspects of their life histories that complicate our ability to reconstruct these phylogenies with confidence.

## Materials and Methods

### Simulations

We constructed an agent‐based simulation written in Golang and available from http://git.sr.ht/~hms/ealdemodor. An initial diploid population is constructed with individuals that consist of one neutral gene with a 1000‐bp sequence. Each diploid individual has two copies of the gene, an age, a maximum age, and a first age at which it reproduces. Each individual is a member of a reproductively isolated population. Individuals within the population share a death rate, a carrying capacity, a branching rate, a meiosis mutation rate, and a somatic/germline mutation rate (referred to as somatic below). The meiosis mutation rate applies at each reproduction event for an individual. The somatic mutation rate is applied at each time step.

At the end of each time step, each population was assumed to be at the maximum carrying capacity. At each generation, individuals were removed randomly, following a random uniform distribution, as in $\left|N\left(0,1\right)\cdot N\right|d$ where *d* is the death rate, and *N* is the population size. For each individual, some branching will result in multiple copies of the genes, as in branching in a biological tree. For each of the genomes within the individual, there was some probability of somatic mutations. If the individual is of reproductive age, it was added to a pool of reproducing individuals. Until the population size reached the carrying capacity, two random individuals were chosen to reproduce. During reproduction, a random gene was chosen from each parent, mutated based on the mutation rate during meiosis, and a new individual was created and added to the population. Simulations were run for 500 000 generations, with population‐splitting events at 166 666 and 333 333 generations. During population splitting, individuals in the speciating population were randomly assigned to one of two new populations, each initially half the size of the original. They reached carrying capacity in the first generation. When a simulation reached the end of the specified number of generations, the gene sequences were written to a file, after which a phylogeny was created for the living individuals and the starting population (for rooting purposes). With this constructed phylogeny, we calculated the average root‐to‐tip distance as a measure of substitution rate for the entire simulation. We calculated conflict in two ways. First, we sampled an individual sequence from each population and then constructed a phylogeny (‘recalculated’). This was replicated 100 times. Second, we sampled one individual from each lineage from the constructed phylogeny of all individuals, keeping the relationships as they were from the constructed phylogeny (‘resampled’). This was replicated 1000 times. In each case, the resulting phylogenies were compared with the known species tree generated by the simulation. We recorded the distribution of ages for each individual at the end of each resulting simulation. We calculated sequence diversity using both *π* (Tajima's theta) and Watterson's theta as: $\pi =\sum_{k=1}^{L}2p_{k}\left(1-p_{k}\right)$ and $\theta_{W}=\frac{S}{\sum_{i=1}^{n-1}\frac{1}{i}}$.

### Simulation runs

Simulations were conducted with the number of generations (500 000), the number of sites (1000), and the birth rate (0.01) remaining constant across runs. Simulations varied or swept the following parameters: meiosis mutation rate, somatic mutation rate, branching rate, death rate, maximum age, time to first reproduction, and carrying capacity (detailed in Supporting Information Tables S1, S2). The number of sites was chosen to be 1000 as is typical for many simulations (e.g. Ren *et al*., 2025). Our simulation mutation rates were such that this length was enough for phylogenetic reconstruction. In all cases, only one parameter was varied, and all others were kept constant to isolate the effect of the variable under study. Each simulation output consisted of a FASTA‐formatted file of terminal gene sequences, along with other output files, to calculate summary statistics. Each parameterization was run for 50 replicates. See Tables S1 and S2 for more details.

### Empirical analyses

We examine several empirical datasets to explore the variation in patterns seen in phylogenomic analyses of long‐lived species. We examine the Fagales and Cucurbitales, the Ericales, the Caryophyllales, and the Gymnosperms. For each of these datasets, we collected data on longevity, phylogenetic gene‐tree conflict, and molecular substitutions. The Fagales, Cucurbitales, and Ericales species‐level phylogenies were obtained from Smith & Brown (2018) and were constructed using Angio353 and GenBank data. Transcriptomic datasets generated as part of Yang *et al*. (2023) were used to estimate gene‐tree conflict rates for each phylogenetic branch of the Fagales and Cucurbitales species trees. Transcriptome datasets generated as part of Carruthers *et al*. (2024) were used to estimate gene‐tree conflict rates for each phylogenetic branch of the Ericales. The Caryophyllales species tree was obtained from Smith *et al*. (2015), and data from Walker *et al*. (2017) were used to estimate gene‐tree conflict on each phylogenetic branch. The Gymnosperm datasets, the species tree, and transcriptomic dataset used to estimate phylogenetic conflict were obtained from Stull *et al*. (2021).

Obtaining maximum longevity estimates poses major challenges for any dataset, but especially for these larger phylogenies. First, there are very few well‐characterized datasets of maximum longevity, with exceptions, such as Loehle (1988). Larger datasets are available through the Botanical Information and Ecological Network (BIEN; Enquist *et al*., 2016; Maitner *et al*., 2018), but their accuracy varies (see Supporting Information Methods S1). Furthermore, the general expectation of accuracy is unclear, as longevity can vary significantly depending on environmental factors, genetics, and other variables. Additionally, how exactly those data are obtained, especially for long‐lived species, adds more error. With all of this in mind, we attempted to gather rough estimates of longevity for our large phylogenies, understanding that there are likely several sources of error. In each empirical example, we attempted to obtain the available data for each species from the BIEN. In total, there were 4231 records for *maximum whole plant longevity* and *longest whole plant longevity*. For species that were not available but for which the genus was sampled in BIEN, we applied the data to each species in that genus. For taxa for which no information was available, we used doomharvest, applied to each genus with three replicates (https://github.com/FePhyFoFum/doomharvest; Supporting Information). Doomharvest uses a public LLM to harvest these or other trait data. Instead of using ages directly, which are prone to being overly precise, we categorized ages into five categories: 0–4, 5–24, 25–99, 100–499, 500+. We compared values from BIEN, Loehle, 1988, and doomharvest to gauge the accuracy of both ages and categories (see Supporting Information; Figs S1, S2). We found that the doomharvest category distribution was normally distributed and like values from BIEN, where 96% of the categories assigned by doomharvest results are either exact or one away from those of Loehle (1988). Similarly, when using BIEN data, 93% of the categories are either exact or one away. Estimated values are available here 10.6084/m9.figshare.29087498.

## Results

### Simulations

**Fig. 1:**
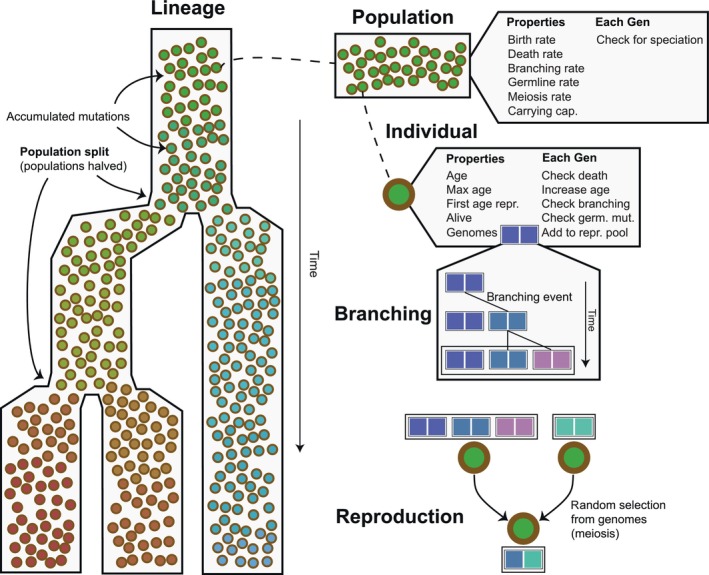
General design of the agent‐based model. The example shows a lineage with two population‐splitting events. Illustrated components include populations and their properties, individuals and their properties, the branching of genomes with mutations, and reproduction, in which haploid genomes are randomly sampled from diploid genomes, undergo meiotic mutation, and are combined.

To examine how lifespan variation shapes evolutionary and population‐level dynamics, we simulated allele patterns under a range of demographic and genetic parameters using forward‐time, agent‐based models (Fig. 1).

**Fig. 2:**
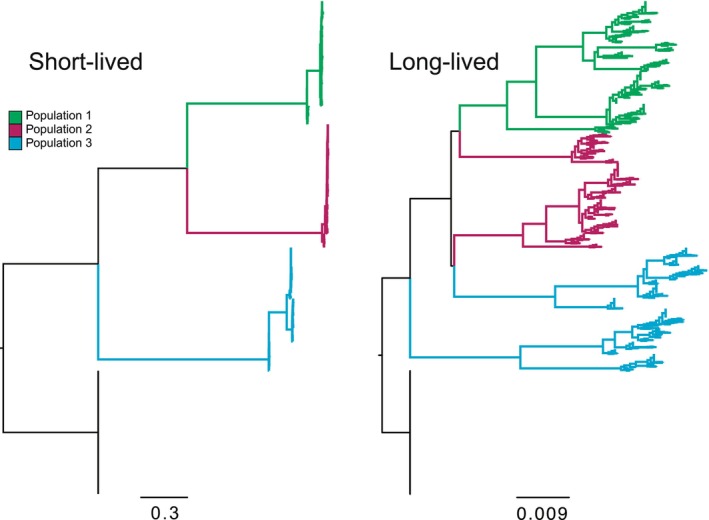
Example phylogenies showing the stark contrast in molecular evolution and tree shape between short‐lived and long‐lived populations. Individuals within the same population are shown in the same color. Long‐lived populations not only exhibit lower substitution rates, but Populations 2 and 3 are also nonmonophyletic.

**Fig. 3:**
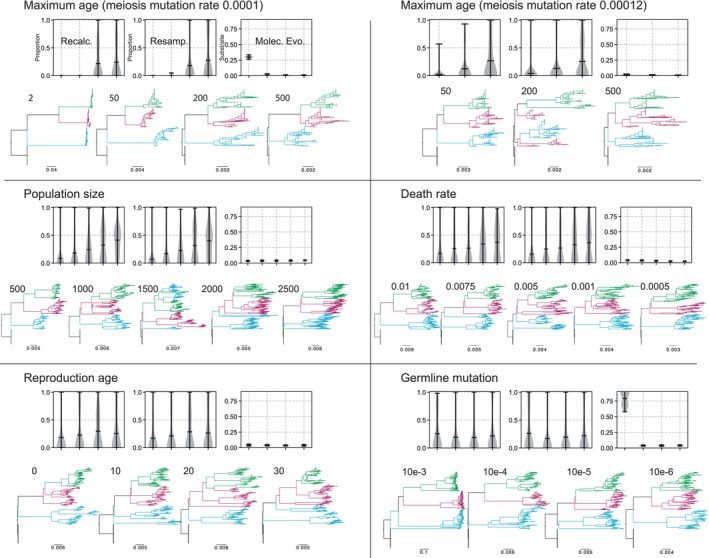
Results from simulation runs, each shown with three metrics: (1) conflict with the species tree when sampling one individual from each population, including the ancestral population, and recalculating the phylogeny (‘Recalc.’); (2) conflict when sampling from the final phylogeny without recalculation (‘Resamp.’); and (3) average tip‐to‐root height as a measure of molecular evolution (‘Molec Evo’). The filled area of the violin plot represents the entire data range. The horizontal line in the plot represents the mean value and the whiskers represent the largest and smallest values.   For parameter details and units see Supporting Information Table S1. The trees and associated numbers below correspond to each entry in the graph on the *x*‐axis. Additional results are provided in the Figs S3 and S4.

We first investigated the effect of maximum individual lifespan on population structure and substitution rates. Populations with shorter lifespans exhibited more rapid generational turnover and faster individual replacement, whereas longer‐lived populations accumulated greater age variance and more persistent genome variants. As maximum lifespan increased from 2 to > 200 yr, the average age at death rose, turnover slowed, and substitution rates declined (Figs 2, 3, S3). However, maximum lifespan extensions beyond 500 yr had diminishing effects, suggesting an asymptotic limit beyond which further longevity no longer alters evolutionary dynamics, given the constant death rates under which data were simulated. Notably, longer lifespans also led to increased conflict between inferred allele trees and the known population genealogy (runs 31–34, *R* ^2^ = 0.91, *P* = 0.045), reflecting the retention of ancestral polymorphism. Interestingly, under high meiotic mutation rates, even long‐lived populations showed substitution rates that were nearly saturated, comparable to annuals (Fig. 2, upper panels; Fig. 3).

Age at first reproduction also influenced genome evolution. Earlier maturation (e.g. at age 0) slightly increased mutation accumulation and substitution rates (from 0.04 with maturation of 0 to 0.035 with maturation at 30), while delayed maturation (e.g. age ≥ 10) slowed population turnover. Gene‐tree conflict was reduced under earlier maturation, from a mean of the proportion of conflicting trees of 0.18 with age 0 at maturation to 0.25 with age at maturation of 30. However, these effects were consistently weak relative to lifespan changes, where conflict increased from 0 to 0.24 with maximum age from 0 to 500 (all with maturation of 10 yr).

Variation in the per‐generation death rate altered the population's turnover dynamics (Fig. S4). Higher death rates (≥ 0.01) increased turnover, reduced average lifespan, and produced younger‐skewed age distributions (see Supporting Information). These conditions also elevated substitution rates (from 0.023 with death rates of 0.0005 and 0.038 with death rates of 0.01) and reduced gene‐tree conflict (halving conflict from 0.36 with death rates of 0.0005 to 0.15 with death rates of 0.01). As death rates decreased, substitution rates plateaued, reaching asymptotes at 0.0001 deaths‐per‐generation‐per‐capita.

Simulations across a range of carrying capacities revealed two distinct and general regimes. Smaller populations (e.g. *n* ≤ 500) exhibited less gene‐tree conflict (0.06 proportion of conflicting trees) due to faster lineage turnover, regardless of time interval. By contrast, larger populations (*n* ≥ 1500) maintained higher levels of standing variation and ancestral polymorphisms and gene‐tree conflict (0.22 proportion of conflict for *n* = 1500 and 0.41 proportion of conflict for *n* = 2500; runs 26–30, *R* ^2^ = 0.98, *P* = 0.001). Substitution rates were not dramatically influenced (from 0.03 to 0.04 with *n* = 500 and 2500, respectively), even with extremely small population sizes, when the maximum age was still high (e.g. 200–500).

Finally, somatic mutation rates had a strong influence on genome divergence. Elevated somatic mutation rates, and especially increased germline mutation rates, greatly amplified substitution rates and sequence diversity. When germline rates exceeded *c*. 0.0001 mutations per generation per individual, they could compensate for the otherwise slow rates observed in long‐lived populations. However, such high rates likely exceed those observed in most empirical systems. It is also important to consider that, as with real systems, the simulations have a ‘spatial’ component to somatic mutations due to the addition of branching. This is not explicitly spatial but instead different branches that may have somatic mutations are isolated from others. This could also impact the results and should be explored further.

### Empirical results

**Fig. 4:**
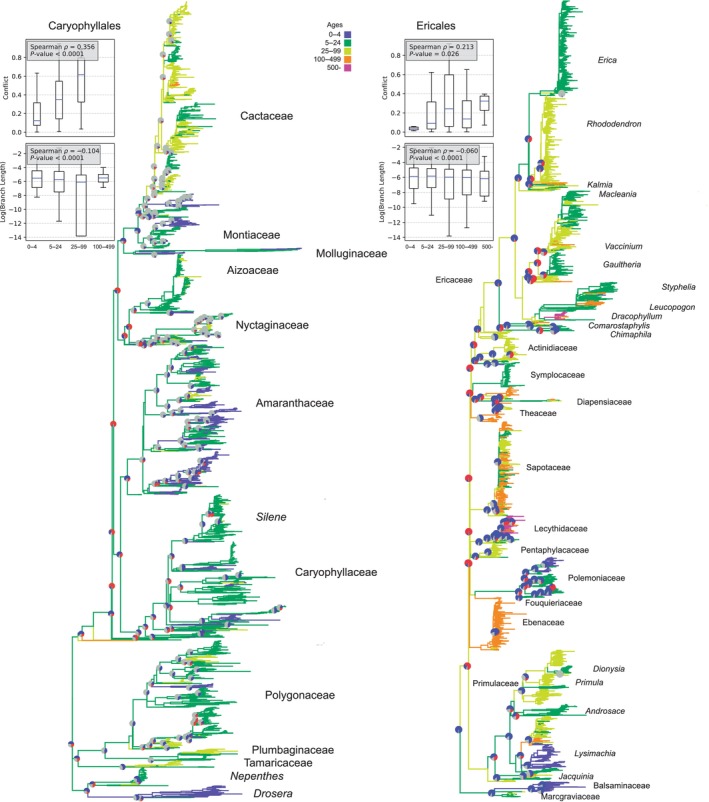
Conflict and molecular evolution in the Caryophyllales (left) and Ericales (right). Pie charts illustrate conflict (red), concordance (blue), and areas of uncertainty due to low bootstrap confidence intervals (gray). The filled area of the violin plot represents the entire data range. The line inside the plot indicates the mean, and the whiskers extend to the smallest and largest values.

We examined life history, molecular evolution, and conflict in four major clades of seed plants: Caryophyllales, Ericales, Fagales, and Cucurbitales, as well as Gymnosperms. The Caryophyllales have life spans ranging from the long‐lived cacti to annuals, such as those in the Nyctaginaceae (Smith *et al*., 2020). The highest conflict in the transcriptomic data occurs within the longer‐lived cacti lineages and along the backbone, which is reconstructed as somewhat intermediate in lifespan (Yang *et al*., 2023). It is essential to note that while longevity has been correlated with shifts in molecular rates, rate changes have also been linked to transitions into different climates within the Caryophyllales (Smith *et al*., 2020). Here, a higher proportion of conflict correlates with longevity (Spearman *ρ* = 0.356, *P* < 0.0001) as well as with lower log substitution rates (Spearman *ρ* = −0.104, *P* < 0.0001; Fig. 4). The Spearman *ρ* for branch lengths is low, but that is to be expected with these large complex clades where certainly many processes are impacting both conflict and rates of molecular evolution; we are likely finding only one of many correlates.

Ericales includes shorter‐lived species, such as *Impatiens*, and several long‐lived species, such as *Pouteria* (Sapotaceae), which can live for several 100 yr. The Ericales also include many long‐lived tree genera, such as those in the Lecythidaceae and *Dracophyllum* (Ericaceae). The backbone of the Ericales has extensive gene‐tree conflict and tends to be reconstructed as a long‐lived tree. We find a correlation between conflict and longevity (Spearman *ρ* = 0.213, *P* = 0.026); short branch lengths (log substitution rates) also correlate with longevity (Spearman *ρ* = −0.060, *P* < 0.0001; Fig. 4). As with the Caryophyllales, Spearman *ρ* for branch lengths is low. The results are similar to those found by Carruthers *et al*. (2024), who examined life history using a broader categorization of habit.

**Fig. 5:**
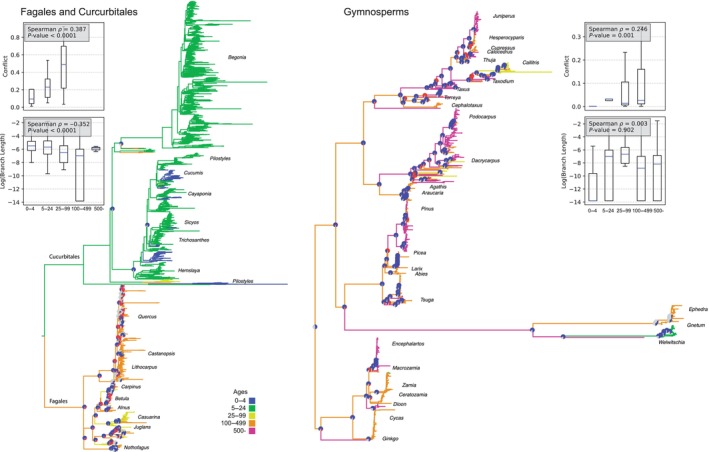
Additional empirical examples of conflict and molecular evolution in the Fagales + Curcurbitales and Gymnosperms. Pie charts illustrate conflict (red), concordance (blue), and areas of uncertainty due to low bootstrap confidence intervals (gray). The filled area of the violin plot represents the entire data range. The line inside the plot indicates the mean, and the whiskers extend to the smallest and largest values.

The Fagales and Cucurbitales demonstrate a major contrast in life history and longevity (Fig. 5). The Fagales contain some of the longest‐lived angiosperm tree species within the flowering plants, and the Cucurbitales include many short‐lived species and annuals. There was a stark contrast in molecular substitution rates between these two orders. While we have fewer Cucurbitales nodes with conflict information than Fagales, the average conflict is much higher in Fagales (0.423) than in Cucurbitales (0.088). We find a strong correlation between conflict and longevity (Spearman *ρ* = 0.387, *P* < 0.0001); short branch lengths (log substitution rates) also correlate with longevity (Spearman *ρ* = −0.352, *P* < 0.0001). While the results are strong within this clade, notably, there are fewer nodes with conflict information available for the herbaceous Curcurbitales.

Gymnosperms include some of the longest‐lived plant species on earth. *Pinus longaeva* (Pinales) can live several 1000 yr, and *Welwitschia mirabilis* (Gnetales) can live > 1500 yr (Fig. 5). By contrast, other Gnetales are relatively short‐lived species, and their branch lengths are much longer than the remainder of the Gymnosperms. Gene‐tree conflicts vary significantly across the clade, with several long‐lived species exhibiting high conflict within the Cupressaceae, Araucariales, and Pinales (average conflict 17% and maximum conflict of 49%). By contrast, the Gnetales have very little conflict, with only one conflicting gene tree. As with the other datasets, we find a correlation between conflict and longevity (Spearman *ρ* = 0.251, *P* = 0.001). However, short branch lengths (log substitution rates) did not correlate with longevity (Spearman *ρ* = −0.008, *P* = 0.725). This test is likely insignificant because there are few nodes with young species (only three taxa with a longevity of < 55 yr).

## Discussion

Our results demonstrate that fundamental aspects of life history can greatly impact phylogenetic conflict patterns and influence expectations of population genetic evolution. Our primary finding, supported by both simulations and empirical data analysis, is that long‐lived species more often exhibit higher conflict and fewer molecular substitutions than short‐lived species. The impact of lifespan variation on conflict and molecular substitution rates depends on several parameters. Extreme longevity results in low substitution and deep divergences within the population, mimicking larger population sizes with higher incomplete lineage sorting (Fig. 2). The primary cause is overlapping generations, where reproductively active long‐lived individuals cause ‘old’ alleles to persist in the population both by their presence in the population and by contributing them to new offspring. If a potentially long‐lived population has higher realized death rates, then its allele and conflict patterns will mimic those of a shorter‐lived population, depending on the death rate and age at maturity. When the age at maturity is older, substitution rates tend to be lower, and conflicts tend to be higher. Somatic mutations and plant branching have the effect of increasing the apparent substitution rate when they are high, although there is still little evidence for high rates of somatic mutations in extant populations. Population size differences have the expected effect of larger populations of long‐lived species having more conflict.

These findings have several implications for the long‐standing discussions about molecular rate heterogeneity in angiosperms (Beaulieu *et al*., 2015; Smith & Beaulieu, 2024). Those conversations have proposed several potential biological mechanisms that might influence generation time and effective population size to explain the differences in molecular evolutionary rates between short‐ and long‐lived species (e.g. DNA repair, metabolic rate, and life history; Martin & Palumbi 1993, Bromham, 2009). In plants, life‐history axes such as annual vs perennial or herbaceous vs woody have been thought to be particularly important in determining evolutionary rates (e.g. Gaut *et al*. 1997, 2011; Andreasen & Baldwin 2001; Smith & Donoghue, 2008; Lanfear *et al*., 2013). While rates of molecular evolution have been suggested to be strongly correlated with life history, the mechanisms underlying this correlation have not been adequately examined (Smith & Donoghue, 2008; Yang *et al*., 2015). The simulations presented here demonstrate how overlapping generations, age‐structured reproduction, and retention of ancestral polymorphisms can reduce the rate at which mutations become fixed, thereby lowering substitution rates. Furthermore, the gene‐tree conflict seen in some long‐lived lineages may be explained by the interaction between speciation time, longevity, and population size. This is consistent with observed gene‐tree discordance in groups, such as Fagales (Hipp *et al*., 2020; Zhou *et al*., 2022) and Ericales (Larson *et al*., 2020; Carruthers *et al*., 2024), where long‐lived lineages exhibit extensive phylogenetic conflict.

These simulations demonstrate that several life‐history and demography parameters can impact phylogenetic patterns. However, these parameters can exhibit strong variation within lineages and even among populations of the same species. This expected variation is clear from the empirical examples, where short branch lengths can exhibit relatively higher conflict (e.g. Ericales and Fagales) or relatively lower conflict (e.g. Gymnosperms and Caryophyllales; Figs 4, 5). Of course, in many of these empirical systems, there are additional factors that can exacerbate and compound these patterns, such as introgression, polyploidy, and hybridization, which are currently not modeled in our simulations. The variation at these large scales likely reflects the extensive variation in life‐history and other properties among lineages. Variation within lineages may be more difficult to detect at larger phylogenetic scales, involving many species and millions of years of divergence. Nevertheless, our simulations suggest patterns we might examine with the right datasets.

Of the several parameters examined, the death rate emerges as one of the most straightforward to interpret. In empirical systems, populations of long‐lived species are likely to vary significantly in death rate due to differences in local disturbance or ecological relationships. We demonstrate in simulations that populations with higher death rates are likely to exhibit patterns more similar to those of shorter‐lived species, with implications for substitution rates, population dynamics, and phylogenetic conflict. In empirical systems, the spatial variation in death rate will complicate these patterns. The impact of integrating populations with spatial variation in death rates on the phylogenetic patterns observed here is not well understood, although these issues have been discussed in relation to age distributions in ecological studies (Wright *et al*., 2003; Petit & Hampe, 2006). Spatial variation in death rate and its effects should be examined further in both empirical and simulation studies.

Another consideration for further examination is the time between speciation events. Here, we demonstrate the impacts of life history on substitution rates and, therefore, branch lengths. However, the time between speciation events will be another significant factor in the realized branch lengths. Along with allele diversity, speciation interval is one of the two classic parameters of incomplete allele sorting (Tajima, 1983; Hudson, 1983), where a longer time between speciation events leads to a lower probability of sampling allele conflict in descendant species. The speciation rate is a population‐ or metapopulation‐level process whose intrinsic and extrinsic causes are generally stochastic and decoupled from the lifespan of organisms. For extraordinarily long‐lived species, one might imagine that a particularly short speciation interval might be on the order of only a handful of maximum lifespan generations. By contrast, a short‐lived species with a long interspecific interval might experience millions of generations between splits. However, the stochastic nature of the appearance of genetic or environmental reproductive isolation barriers means that the variance provides only a loose ceiling on this pattern. Therefore, the relative timing of generations, lifespan, and speciation events may exert a significant influence on phylogenetic patterns, as demonstrated here.

While we have addressed some of the standing questions facing life‐history and molecular evolution, many more remain unanswered. For example, the simulations only include two evenly spaced population‐splitting events to simplify the model, resulting in three final reproductively isolated populations. The frequency and rate of population splitting both have a significant impact on phylogenetic patterns and could be explored further in conjunction with the parameters examined in this study. Furthermore, populations immediately return to carrying capacity after splitting events and reproduction ceases between sister populations (e.g. no introgression). Future studies could examine the impacts of additional complexity in relaxing these assumptions. For the purposes of this study, however, we expect introgression, for example, to exacerbate the patterns we observe. We also only consider populations that share life‐history traits. This was motivated to isolate the impacts of individual parameters on molecular variation. However, life‐history shifts occur across the plant tree of life at varying frequencies. The effects of these changes on the patterns of rate variation and gene‐tree conflict require additional examination. Within population dynamics, further exploration could also be conducted, such as age structure (e.g. Cannon *et al*., 2022). Additionally, we examine only a 1000‐bp gene region. To better reflect our data collection efforts, expanding the number of gene regions to hundreds or thousands would be enlightening. Each extension can be implemented within the current framework discussed in the methods and our code. Other future axes of complexity beyond the simulation framework would also be fascinating to explore, such as spatially explicit patterns of molecular variation.

This study presents an agent‐based model to explore the impacts of life history and population dynamics on molecular evolution. Mechanisms exist to examine certain components and variables within a more traditional mathematical framework of population genetics. However, those traditional frameworks are limited in fully accommodating the complexity of some of the elements explored here. For example, while the literature on life history and molecular evolution frequently discusses longevity, a potential outcome of longevity, overlapping generations, is rarely considered. Additionally, the potential for somatic mutations that may accumulate over a long‐lived plant's lifetime to contribute to a population's molecular evolution adds a significant layer of complexity. Agent‐based models enable the unconstrained development of models with realistic parameterizations. Of course, this, in turn, comes with its challenges, as the added complexity may overwhelm the focus of the question at hand. Striking a balance between adding variables that are essential to addressing the hypotheses in question and adding superfluous variables can be a difficult task.

Many extensions to the modeling framework await exploration. However, our findings also highlight facets of available data that are underexplored. Genomics datasets range from those with species‐poor coverage and broad sampling to those with extensive resampling of a single species. Several projects have aimed to expand the sampling of genomic datasets greatly, and these resources will undoubtedly become available. However, in the meantime, several barriers exist to using these data for addressing some of the questions explored here. Datasets focusing on population genetics are invaluable for exploring phylogeographic patterns and recent population dynamics. However, these datasets are often too limited in geographic or phylogenetic scope to capture transitions in life history or other complexities. By contrast, systematic datasets rarely sample more than one individual for each species. An exciting avenue lies in waiting for datasets that span these typical sampling boundaries.

While exploring and extending these simulations will provide additional insights into the impacts of life history on broad plant evolution, more in‐depth empirical analyses would also be essential. Unfortunately, and as discussed in the methods and supplement, data on maximum longevity are particularly scarce for both genera and species. We aimed to overcome this by employing a diversity of data collection techniques, including AI tools. The results from these data collection efforts require scrutiny and cannot, at least in our exploration, be used directly. Nevertheless, in areas where data are scarce, we found utility in augmenting existing data with these sources. Efforts to improve these datasets, along with the inherent uncertainties in the data, would be highly fruitful for furthering our understanding of the evolution of life histories.

## Competing interests

None declared.

## Author contributions

SAS wrote the code and conducted analyses. SAS, JBP and JMB initially conceived the study. JBP and GSB assisted with the population and simulation analyses. T‐SY, YY, GWS and TC helped analyze the empirical datasets. IBH and WNW assisted with data collection and doomharvest processing. All authors contributed to the manuscript.

## Disclaimer

The New Phytologist Foundation remains neutral with regard to jurisdictional claims in maps and in any institutional affiliations.

## Supporting information


**Fig. S1** Age estimate and the category estimate comparison.
**Fig. S2** Age estimate and the category estimate comparison with species.
**Fig. S3** Simulation age distributions under various maximum ages.
**Fig. S4** Simulation age distributions under various death rates.
**Methods S1** Aggregating life‐history data with doomharvest.
**Table S1** Description of simulation parameters.
**Table S2** Simulation run parameters.
Please note: Wiley is not responsible for the content or functionality of any Supporting Information supplied by the authors. Any queries (other than missing material) should be directed to the *New Phytologist* Central Office.

## Data Availability

The data that support the findings of this study are available at doi: 10.6084/m9.figshare.29087498. Other data have been previously published and cited in the text.
